# Embryonic Development in Relation to Maternal Obesity Does Not Affect Pregnancy Outcomes in FET Cycles

**DOI:** 10.3390/healthcare10040703

**Published:** 2022-04-10

**Authors:** Adham Fawarseh, Yuval Atzmon, Nardin Aslih, Asaf Bilgory, Einat Shalom-Paz

**Affiliations:** 1 Rappaport Faculty of Medicine, The Technion-Israel Institute of Technology, Haifa 32000, Israel; adhamfaw@gmail.com; 2 IVF Unit, Department of Obstetrics & Gynecology, Hillel Yaffe Medical Center, Hadera 38100, Israel; atzmony@gmail.com (Y.A.); nardin_aslih@yahoo.com (N.A.); asaf_bil@hotmail.com (A.B.)

**Keywords:** BMI, frozen embryo transfer, embryo morphokinetics, pregnancy rate

## Abstract

This retrospective cohort study examined the effect of maternal BMI on embryo morphokinetics using a time-lapse incubator (TLI) and evaluated the effect on outcomes of frozen embryo transfer (FET) cycles. The study included 641 women who underwent FET of a total of 2553 embryos from January 2017 to August 2019. The women were divided into four groups according to BMI: underweight (<18.5 kg/m^2^), normal weight (18.5–24.99 kg/m^2^), overweight (25.0–29.99 kg/m^2^), and obese (≥30 kg/m^2^). Embryos were transferred on day 3 or 5, and their development was monitored using a TLI. We found that oocytes from obese patients were slower in the extraction of the second polar body (tPB2) after fertilization and the two pronucleus stage appeared later compared to normal-weight women. The time to fading of the pronucleus (tPNf), t2, and t4 were comparable between the four groups. Oocytes from underweight and overweight women had significantly faster cleavage at t3 and t5–t8 compared to normal weight. We did not find any significant difference in pregnancy rate, clinical pregnancy rate, or miscarriage rate among groups. In conclusion, embryos from normal-weight patients had slower cleavage rates compared to obese patients, while embryo quality was similar between BMI groups. The cycle outcomes demonstrated comparable pregnancy rates among the BMI groups.

## 1. Introduction

Obesity is a chronic disease with increasing prevalence globally. Among women, 40% are considered overweight and 15% obese. It was estimated that 25% of pregnancy complications (e.g., preterm birth, gestational diabetes, preeclampsia) are attributed to maternal weight and obesity [1]. Obesity and overweight are associated with significantly lower live-birth rates and increased miscarriage rates following IVF treatments [2]. Even in the case of normal menstrual cycles, the time to conception for obese women is longer than it is for normal-weight women due to changes in the menstrual cycle and disturbances to the hypothalamic–pituitary axis [3].

An increase in BMI may affect IVF in various ways, including impaired oocyte quality, which leads to poorer embryo quality, lower implantation rates, and higher early-miscarriage rates [4,5,6]. It is also known that obesity impairs ovarian responsiveness to medications; therefore, these women need larger doses of gonadotropins [4,5,6,7].

With the goal of obtaining the best pregnancy rate per transfer, great efforts have been expended on defining the best embryo quality [8,9,10]. Morphokinetics, using a time-lapse incubator (TLI) system, is used to assess an embryo based on more information than is obtained from traditional methods, in which the embryologist assesses its quality under a microscope. In a TLI, embryos are observed, and images are captured automatically at set time intervals. A TLI minimizes changes in the culture environment during development by maintaining a stable temperature, pH, humidity, and gas concentration. Another advantage is achieved by monitoring the dynamic morphology and identifying abnormal developmental patterns that can help detect embryos with the best growth potential [11,12,13]. Correlations between BMI, embryo morphokinetics, and FET treatment outcome have not been evaluated.

The study evaluated the contribution of embryo morphokinetics on FET outcomes based on the impact of BMI.

## 2. Methods

In this retrospective cohort study conducted at the IVF Unit in Hillel Yaffe Medical Center, records of all patients who underwent FET from January 2017 to August 2019 were evaluated. Inclusion criteria were the use of time-lapse technology to assess morphological and morphokinetic developmental patterns during fresh embryo development. Only autologous oocytes were included. Clinical outcomes were derived from 641 patients and their 2553 embryos transferred on day 3 or day 5.

The body mass index (BMI) of patients included in the study was calculated. BMI is defined as weight in kilograms divided by the square of the height in meters (kg/m^2^). Participants were divided into 4 weight groups according to the most recent World Health Organization classification of BMI categories. BMI < 18.5 kg/m^2^ (*n* = 43) defined as underweight, BMI = 18.5–24.99 kg/m^2^ (*n* = 286) defined as normal weight, BMI = 25.0–29.99 kg/m^2^ (*n* = 154) defined as overweight, and BMI ≥ 30 kg/m^2^ (*n* = 158) defined as obese [14].

### 2.1. Endometrial Preparation Protocols for FET Cycles

Endometrial preparation for the FET and the choice of a treatment protocol, type, and doses of medication were prescribed on a case-by-case basis, according to patient characteristics and clinician preferences and judgment. Dose adjustments were performed according to endometrial response, which was monitored by transvaginal ultrasound (TVUS) scans. The doses were adjusted according to endometrial thickness only and not BMI. Estradiol blood levels were measured in natural cycles.

### 2.2. FET in Ovulatory Cycles

TVUS examination was performed during menstruation and again on the eighth to the tenth days of the cycle. When the diameter of dominant follicles was ≥18 mm, the estradiol level was decreased compared to the previous day, and luteinizing hormone (LH) was markedly increased. This was considered ovulation day, and the transfer was planned according to the embryo’s age. On the day of the transfer, embryo or blastocyst transplantation was conducted using abdominal ultrasound.

### 2.3. Artificial FET Cycle

On the third day of menstruation, TVUS was performed to evaluate the endometrial thickness and ovarian status. Oral estradiol valerate (Progynova, Bayer HealthCare, Germany or Estrofem Novo Nordisk a/S, Denmark) was administered at 2 mg daily. TVUS was performed again 7 days later, and the hormone dose was adjusted depending on the endometrial thickness.

### 2.4. Evaluation of Time-Lapse Images

Images of the embryos were acquired with EmbryoViewer software (Unisense FertiliTech, Aarhus, Denmark). Cleavage time was considered the moment when cell division was completed, and the two originating cells were completely segregated and enclosed in their respective cytoplasmic membranes. The time points assessed in the study were defined as tPB2, time to second polar body extraction; tPNa, time to pronucleus appearance; tPNf, time of pronucleus fading; and t2, t2, t3, t4, t5, t8 represent the time points when the blastomere embryo reached the stages of 2, 3, 4, 5, and 8 cells, respectively. The time point t8 was the last parameter assessed, even for embryos further cultured to be transferred on day 5. Other parameters analyzed were Cc2 and Cc3 (intervals between t2–t3 and t3–t5, respectively), reflecting the duration of the second and third cell cycles, and s2 and s3, reflecting the synchrony of cell divisions from two to four blastomeres (s2) and five to eight blastomeres (s3). Embryo quality was calculated based on the KIDS score using the time-lapse model collecting all the above data, as described in the Istanbul consensus. The KIDS is a model based on morphology and morphokinetic traits associated with the implantation potential of embryos by Vitrolife^®^, Ottawa, ON, Canada [15].

### 2.5. Outcome Measures

The primary outcome was embryo morphokinetics, based on the time points noted above. Secondary outcomes were embryo quality based on KIDS score, ongoing pregnancy, and spontaneous abortion. The primary parameters used to assess the embryo quality score were discussed in the Istanbul Consensus [15].

The β-hCG level was measured 14 days after embryo transfer. TVUS was performed 2 weeks after a positive pregnancy test. Clinical pregnancy was confirmed when a gestational sac with a fetal heartbeat was visible by ultrasound examination after 6 weeks of pregnancy.

Data collected included baseline parameters (age, parity, cause of infertility, BMI, basal FSH, and LH), cycle characteristics (treatment protocol, endometrial thickness, number of transferred embryos, and embryo quality), and cycle outcomes (chemical pregnancy, clinical pregnancy, live birth rates, and miscarriage rate). Demographic data, treatment information and results, and pregnancy outcomes were recorded and followed until delivery.

### 2.6. Statistical Analysis

Statistical analysis was performed using SPSS, version 25 (SPSS Inc., Chicago, IL, USA). We used the Shapiro–Wilk test to evaluate the distribution of the data. When appropriate, comparisons were analyzed using a Student’s *t*-test or Mann–Whitney U test. Proportions were compared using a Chi-Square test or Fisher exact test.

The sample size calculation was based on the assumption that: (1) Each of the BMI subgroups was compared only to the normal BMI group. (2) Power was set at 80% with a 95% confidence interval. (3) Normal BMI (18.5–24.99) had a higher pregnancy rate compared to the overweight and obese subgroups (40% vs. 25%, respectively). Based on these assumptions, we needed to recruit 155 women in each group. https://www.openepi.com/SampleSize (accessed on 8 March 2022) version 3 was used to calculate the sample size.

Baseline characteristics of the BMI groups were compared using the Mann–Whitney U-test. Normal BMI was considered as the reference group. Kruskal–Wallis test was used to compare the 4 groups, and a post hoc comparison compared each group to the control group using Dunn’s test. We used a logistic regression model to assess the effect of BMI and other demographic and clinical parameters to predict clinical pregnancy. We adjusted for the following potential confounders: maternal age, endometrial thickness, BMI subgroups, and KID scores. P-values less than 0.05 were considered significant.

## 3. Results

We evaluated a total of 641 patients treated with different FET protocols and 2553 embryos. They were divided into four BMI groups, group A < 18.5 kg/m^2^ (*n* = 43) defined as underweight, group B = 18.5–24.99 kg/m^2^ (*n* = 286) defined as normal weight, group C = 25.0–29.99 kg/m^2^ (*n* = 154) defined as overweight, and group D ≥ 30 kg/m^2^ (*n* = 158) defined as obese. All the results and statistical analysis were compared to group B (normal weight) as the main reference group.

Patients’ characteristics are presented in Table 1. The baseline characteristics of the four groups were similar, including patients’ age, FSH, LH, gravidity, and parity. Treatment protocols per group were also comparable, as was endometrial thickness before the transfer.

Embryo quality and treatment outcomes are presented in Table 2. A comparable number of embryos was transferred per patient in all four groups. Embryos at the cleavage stage or blastocyst stage were transferred equally among the groups. Embryo morphology, as assessed by KID scores, did not vary significantly across BMI groups. Embryo quality and FET pregnancy outcomes were comparable. The miscarriage rate was also similar among the groups.

Morphokinetic parameters according to the embryoscope are presented in Table 3. Group D had longer tPB2 and tPNa compared to group B. Moreover, tPNf, t2, and t4 between the four groups were similar. Groups A and C had significantly faster cleavage at time points t3 and t5–t8.

We conducted a multivariate analysis to evaluate which parameters affected pregnancy rates (Table 4). The following parameters were included: BMI as a categorical variable, maternal age, endometrial thickness on the last day of evaluation before the transfer, and quality of embryos available for transfer.

We found that the underweight group (BMI < 18.5) had a lower chance of conception, with an odds ratio (OR) of 0.063, 95% CI 0.005 to 0.739; *p* = 0.028. Other groups did not reach significance. Advanced maternal age and higher embryo quality had an influence on pregnancy rates (OR for each additional year of maternal age 0.85, 95% CI 0.755 to 0.958; *p* = 0.008 and for better embryo quality OR 2.754, 95% CI 1.179 to 6.43; *p* = 0.019).

## 4. Discussion

This study examined the effect of BMI on FET outcomes based on embryo morphokinetics obtained from time-lapse technology. We chose to evaluate FET cycles and the available frozen embryos per patient to minimize confounding effects and eliminate the impact of fresh treatment parameters on cycle outcomes, such as doses of gonadotropins, supra-physiologic hormonal level in fresh cycles, risk of ovarian hyperstimulation syndrome, etc. We also chose the best available embryos, all of which underwent the same freezing method.

We found that among obese patients (group D), extraction of the second polar body (tPB2) after fertilization was slower, and the two pronucleus stages appeared later in oocytes compared to women with normal BMI. The time to fading of the pronucleus (tPNf), t2, and t4 were comparable between the four groups. Groups A and C had significantly faster cleavage at time points t3 and t5–t8 compared to normal-weight women. We did not find any significant differences in outcome parameters of pregnancy rate, clinical pregnancy rate, and miscarriage rate, although some trends were noticed.

This study reports no significant differences between women with BMI ranging from underweight to obese in terms of embryo quality, cycle treatment outcomes, and pregnancy results. However, it is important to mention the potential differences raised in the literature and in this study regarding oocyte and fertilized oocyte performance.

The literature is inconsistent regarding the impact of obesity on reproductive outcomes, fecundability, and IVF outcomes. Some studies suggested no negative effect on clinical outcomes between obese and non-obese patients [6,16,17], while others reported impairment in several parameters, including implantation rate, clinical pregnancy rate [2,18,19], live birth rate [2,18,19], and miscarriage rate [2]. The effect of obesity on oocyte and embryo quality is also inconsistent. Some reports found lower embryo quality in obese patients undergoing IVF [20], while others reported no adverse effects from obesity on oocyte and embryo quality [21]. The results of these studies indicate that embryos of normal-weight patients had slower cleavage rates compared to those of obese and underweight patients. This suggests that the classical morphological parameters routinely used to assess embryo quality may not be accurate enough to detect the potential effects of maternal BMI on oocyte and embryo development. It is possible that previous studies did not reach any conclusions regarding maternal obesity and embryo development because they used static morphology assessment. Considering the limitations of previous studies, we evaluated embryo development using a TLI system. This technology was shown to improve embryo selection for transfer [22].

Our results do not agree with those of Bartolacci et al. [23], who reported that embryos from overweight and obese women had slower cleavage rates, with significant differences at t5 and t8. Our results also contradict those of Bellver et al. [24], who reported that embryos from obese and normal-weight patients had similar cleavage patterns. However, overall, our study and those of Bartolacci [23,24] agree about the similarities in embryo quality scores among BMI groups.

### 4.1. Morphokinetics and Embryo Quality of Obese and Normal-Weight Patients

Our study demonstrated that the embryo quality on the day of transfer was comparable between groups. This may be attributed to the decision to cryopreserve the best embryo and that all were preserved using the same freezing method.

Our results support the findings of similar final embryo quality suitable for cryopreservation among women with different BMI [6,21]. However, the morphokinetic parameters seemed different, with significantly faster development in the early stage of fertilization and significantly slower during the cleavage stage in normal-weight patients compared with obese women.

It was proposed that the final morphological parameters of the embryos can be used along with morphokinetic parameters to predict embryo quality and development [25,26]. Most studies examined embryo morphology, while few studies compared morphokinetics between different BMI groups. A meta-analysis by Roque et al. [27] showed that frozen embryos had significantly higher ongoing pregnancy and clinical pregnancy rates compared to fresh embryos. They suggested this was due to the impairment of endometrial receptivity caused by controlled ovarian hyperstimulation [28], and for fresh embryos, elevated progesterone levels may cause asynchrony between the endometrium and embryo development. These results were mainly attributed to the endometrium. The effect of embryo morphokinetics and morphology were not taken into consideration. Based on this assumption, we aimed to evaluate pregnancy outcomes in four different BMI groups using top-quality embryos that were cryopreserved for future FET cycles. This enabled us to overcome the confounding influence of supra-physiologic hormone levels found in fresh cycles.

### 4.2. Does BMI Affect Cleavage Rate?

#### Fast Cleavage vs. Slow Cleavage

Several studies evaluated the differences between fast and slow cleaving embryos. Embryos from fast- and slow-dividing groups exhibited different metabolic parameters, including pyruvate and lactate metabolism, glucose consumption, and glutamate metabolism. Glycogen storage was higher in the blastocysts in the slow-dividing group. On the other hand, blastocysts in the fast-dividing group had a higher concentration of lipids [29] and significantly fewer mitochondrial DNA (mtDNA) [30].

Our study demonstrated that the oocytes from normal-weight women completed the second meiosis significantly faster than those from obese patients. However, cell division throughout the cleavage stage in t5–t8 in oocytes from normal-weight patients was slower. There is a consensus among researchers that embryo kinetic parameters are predictive of the likelihood of achieving pregnancy. Our data support the assumption that slower cell division correlates with a longer time for cell repair, which results in higher-quality embryos. Motato et al. [31] timed cell cycles and found that the duration of the second and third cell cycles was longer in embryos that developed to blastocysts, as compared with embryos arresting before the blastocyst stage. This may indicate that these cell stages require a minimum period for orderly, efficient DNA synthesis as part of the cell cycle.

Desai et al. [32] found that specific, early cell cycle kinetics were predictive of an embryo’s ability to develop to the blastocyst stage but did not correlate to embryo ploidy. In contrast, late kinetic parameters, slower cleaved embryos, appeared to be associated with a greater likelihood of euploidy. This may explain the trend towards better pregnancy and lower miscarriage rates in the group of normal-weight women, in which slower cleavage was observed.

The contribution of this study is that it evaluated pregnancy rates using frozen embryos only, thereby controlling the confounders that may impact pregnancy rates during fresh cycles, such as supra-physiologic hormone levels [27].

On the other hand, we included all the embryos from each patient’s fresh cycle in the morphokinetic analysis. This enabled us to understand the impact of BMI on embryo development and morphokinetics and led to optimal conditions for embryo transfer.

This study is limited by its retrospective nature and the fact that it was conducted in a single IVF center. However, the large number of embryos analyzed in all BMI groups strengthens the soundness of the study.

In conclusion, the current study indicates that embryos from normal-weight patients have a slower cleavage rate compared to those from obese patients, which agrees with previous studies, where embryo quality was similar, and cycle outcomes for the normal weight group were better, although not significantly so. Choosing slower dividing embryos may be associated with better embryo quality.

Additional studies with a larger sample size are recommended to evaluate the effect of maternal BMI on embryo morphokinetics and IVF outcomes.

## Figures and Tables

**Table 1:** Patients’ characteristics according to BMI group.

Characteristic	Group A (<18.5) *n* = 43	Group B (18.5–24.9) *n* = 286	Group C (25.0–29.9) *n* = 154	Group D (≥30) *n* = 158
BMI	17.6 ± 0.65	21.9 ± 1.80	27.1 ± 1.6	33.5 ± 3.5
Age, years	33.3 ± 7.2	34.9 ± 6.2	35.9 ± 7.4	36.1 ± 5.2
Cause of infertility (%)				
Unexplained	2	7	9	6
Male factor	44	50	42	44
Anovulation	2 *	15	13	30 *
Mechanical	26	18	23	23
Endometriosis	**0**	**2**	**5**	**1**
FSH, IU/L (Baseline)	5.84 ± 2.4	7.91 ± 2.67	10.91 ± 5.87	6.86 ± 1.79
LH, IU/L (Baseline)	6.9 ± 2.31	8.2 ± 2.9	6.8 ± 2.6	7.6 ± 3.5
Gravidity 0 1 or more	13 (30%) 30 (70%)	88 (31%) 197 (69%)	39 (25%) 115 (75%)	42 (27%) 116 (73%)
Parity 0 1 or more	29 (67%) 14 (33%)	154 (54%) 131 (46%)	79 (51%) 75 (49%)	80 (51%) 78 (49%)
Previous transferred cycles	4.9 ± 2.8	4.3 ± 2.9	3.9 ± 2.2	4.4 ± 2.3
Smoker	5 (15%)	51 (20%)	25 (21%)	24 (18%)
Type of transfer protocol				
Artificial cycle (E + P)	25 (58%)	145 (51%)	88 (58%)	90 (58%)
Ovulatory cycle	18 (42%)	137 (49%)	65 (42%)	66 (42%)
Endometrial thickness before transfer (mm)	8.62 ± 1.5	9.13 ± 2.18	8.67 ± 2.06	9.10 ± 2.52

* *p* < 0.05, in comparison with group B (normal BMI).

**Table 2:** Cycle parameters and results based on maternal BMI groups.

Parameter	Group A (<18.50) *n* = 43	Group B (18.50–24.99) *n* = 286	Group C (25.0–29.99) *n* = 154	Group D (≥30) *n* = 158
Number of embryos transferred	1.16 ± 0.37	1.25 ± 0.43	1.23 ± 0.42	1.22 ± 0.41
Completed cycles n (%)	43 (100%)	281 (98%)	152 (99%)	153 (97%)
Cleavage stage embryos transferred (%)	16/21 (76%)	103/159 (65%)	59/77 (77%) *	61/93(66%)
Blastocyst stage embryos transferred (%)	5/21(24%)	56/159 (35%)	18/77 (23%)	32/93 (34%)
Total KID for all available embryos	4.81 ± 0.40	4.59 ± 0.73	4.72 ± 0.46	4.58 ± 0.63
Positive pregnancy test per transfer	13 (31.0%)	123 (46.4%)	57 (41.6%)	61 (43.6%)
Clinical pregnancy per transfer	12 (27.9%)	108 (37.8%)	48 (31.2%)	49 (31.0%)
Miscarriage	1/43 (2.3%)	15/286 (5.2%)	9/154 (5.8%)	12/158 (7.6%)

* *p* < 0.05, in comparison with group B (normal BMI).

**Table 3:** Morphokinetic parameters (hours) of all available embryos according to BMI group.

Parameter	Group A (<18.50) *n* = 239	Group B (18.50–24.99) *n* = 960	Group C (25.0–29.99) *n* = 650	Group D (≥30) *n* = 704
tPB2	2.72 ± 1.07 *	2.95 ± 1.49	3.17 ± 2.56	3.08 ± 2.51 *
tPNa	6.54 ± 1.41	6.81 ± 2.48	6.91 ± 2.24	7.15 ± 2.84 *
tPNf	24.78 ± 5.02	25.05 ± 4.54	24.67 ± 5.13	24.96 ± 6.06
t2	27.31 ± 4.89	27.72 ± 4.66	27.43 ± 5.46	27.73 ± 6.09
t3	36.96 ± 5.33 **	38.18 ± 6.32	37.35 ± 6.47 *	38.18 ± 6.56
t4	39.63 ± 6.47	40.42 ± 6.75	40.35 ± 7.63	40.80 ± 7.71
t5	49.29 ± 7.56 *	50.99 ± 8.59	50.00 ± 8.98 *	51.41 ± 9.00
t6	52.28 ± 7.43 **	54.31 ± 8.70	53.16 ± 9.25 *	54.96 ± 9.23
t7	55.66 ± 9.31 **	57.57 ± 9.85	56.03 ± 9.71 *	57.65 ± 9.73
t8	58.26 ± 10.05 **	60.88 ± 11.19	59.38 ± 11.00 *	60.93 ± 11.29
Cell cycle				
Cc2	9.88 ± 4.31 *	10.65 ± 4.54	10.15 ± 4.88 *	10.70 ± 4.63
Cc3	12.65 ± 5.58	13.25 ± 5.84	13.26 ± 6.46	13.65 ± 6.39
s2	2.72 ± 4.88	2.40 ± 4.25	3.10 ± 5.58	2.83 ± 5.25
s3	9.83 ± 8.97	10.67 ± 9.64	10.27 ± 9.65	10.87 ± 9.68

* *p* < 0.05, ** *p* < 0.01, in comparison with group B (normal BMI).

**Table 4:** Multivariate analysis of parameters affecting pregnancy rates.

	Odds Ratio	Significance	95% CI Lower	95% CI Lower
BMI Group A	0.063	0.028	0.005	0.739
BMI Group C	0.731	0.712	0.139	3.858
BMI Group D	1.439	0.622	0.339	6.104
Maternal Age (Years)	0.851	0.008	0.755	0.958
Endometrial thicjness on HCG day	1.024	0.882	0.750	1.399
Embryo Quality based on ESHRE criteria	2.754	0.019	1.179	6.431

## Data Availability

Data will be made available upon request.
